# Motor cortical neuronal hyperexcitability associated with α-synuclein aggregation

**DOI:** 10.1038/s41531-024-00867-z

**Published:** 2025-01-15

**Authors:** Liqiang Chen, Hiba Douja Chehade, Hong-Yuan Chu

**Affiliations:** 1 Aligning Science Across Parkinson’s (ASAP) Collaborative Research Network, Chevy Chase, MD 20852 USA; 2 Department of Neurodegenerative Science, Van Andel Institute, Grand Rapids, MI 49503 USA; 3 Department of Pharmacology and Physiology, Georgetown University of Medical Center, Washington, DC 20007 USA

**Keywords:** Parkinson's disease, Cellular neuroscience

## Abstract

In Parkinson’s disease (PD), Lewy pathology deposits in the cerebral cortex, but how the pathology disrupts cortical circuit integrity and function remains poorly understood. To begin to address this question, we injected α-synuclein (αSyn) preformed fibrils (PFFs) into the dorsolateral striatum of mice to seed αSyn pathology in the cortical cortex and induce degeneration of midbrain dopaminergic neurons. We reported that αSyn aggregates accumulate in the motor cortex in a layer- and cell-subtype-specific pattern. Specifically, αSyn aggregates-bearing intratelencephalic neurons (ITNs) showed hyperexcitability, increased input resistance, and decreased cell capacitance, which were associated with impaired HCN channel function. Morphologically, the αSyn aggregates-bearing ITNs showed shrinkage of cell bodies and loss of dendritic spines. Last, we showed that partial dopamine depletion is not sufficient to alter thalamocortical transmission to cortical pyramidal neurons. Our results provide a novel mechanistic understanding of cortical circuit dysfunction in PD.

## Introduction

Degeneration of dopaminergic neurons in the substantia nigra pars compacta (SNc) has been associated with accumulation of cytoplasmic Lewy-like pathology in most PD cases^1^. Reduction of dopamine (DA) levels in the basal ganglia significantly alters the connectivity and computation of the basal ganglia-thalamocortical circuits in parkinsonism. The traditional circuit model of PD pathophysiology suggests that the aberrant inhibitory output of the basal ganglia disrupts the thalamic excitation to the motor cortex, which decreases cortical motor output, underlying the hypokinetic symptoms in PD^2–5^. This model assumes that cortical microcircuits remain intact in parkinsonian state and that cortical hypofunction is an instant effect of the exaggerated suppression of the thalamus by the basal ganglia. However, converging evidence from recent studies suggests that the motor cortex shows intrinsically disrupted connection and function in animal models of parkinsonism that can play a major role in the cortical pathophysiology of PD^6,7^. These local cortical changes include altered dendritic spine dynamics and density of cortical pyramidal neurons and reduced thalamic axonal markers in the primary motor cortex (M1) of both parkinsonian monkeys and rodents^8–10^. Such anatomical changes are also associated with altered thalamocortical connection strength and dampened intrinsic excitability of pyramidal tract neurons (PTNs) in M1^8,11,12^. Consistently, in vivo studies also reported a disrupted timing and magnitude of M1 PTNs activation in mediating motor activities in parkinsonian animals^13–15^. Thus, the motor cortex is a site of intrinsic dysfunction instead of being an information gateway that translates basal ganglia abnormalities into motor deficits in PD^7^.

Most prior research on cortical dysfunction has been conducted using dopamine-depleted neurotoxin models of parkinsonism that usually do not develop Lewy-like pathology. Post-mortem studies of human PD reported moderate levels of Lewy pathology in cerebral cortical motor regions at the Braak stages 4–6, indicating a potential role of cortical pathology in motor and cognitive impairments in PD^16–19^. However, how the development of Lewy pathology disrupts motor cortical circuit integrity and function remains largely unexplored. In the present study, we studied the functional impact of α-synuclein (αSyn) aggregates on motor cortical neurons and their synaptic inputs using αSyn preformed fibrils (PFFs)-seeding model of synucleinopathy. We demonstrated that αSyn aggregates triggered cellular hyperexcitability of intratelencephalic neurons (ITNs) in the secondary motor cortex (M2) in association with increased input resistance and decreased cell capacitance. The hyperexcitability of ITNs was partially abolished by pharmacological blockade of HCN channels. Congruous with physiological alterations, αSyn aggregates-bearing ITNs in the M2 showed more packed cell bodies, reduced dendritic length, and loss of dendritic spines. Moreover, we found that mild loss of striatal dopamine associated with αSyn aggregation is insufficient to induce changes in the intrinsic and synaptic properties of corticospinal neurons (CSNs) in M2. These results demonstrate that the intrinsic excitability of cortical pyramidal neurons is enhanced by the accumulation of intracellular αSyn aggregates and that severe striatal dopamine loss is required to induce adaptive circuit changes in the motor cortex in parkinsonism.

## Materials and methods

### Animals

Three-to-four months old wild type male C57BL/6 J mice (JAX stock#:000664, RRID: IMSR_JAX:000664) were used in this study and were provided by the Van Andel Research Institute vivarium. Mice were housed up to four animals per cage under a 12 h/12 h light/dark cycle with free access to food and water following NIH guidelines for animal care and use. Animal studies were reviewed and approved by the Institutional Animal Care and Use Committee (IACUC) at Van Andel Institute (animal use protocol #: 22-02-007).

### Preparation and validation of αSyn preformed fibrils

Escherichia coli BL21 codon plus RIPL cells (RRID: CVCL_M639) were used to produce and purify mouse αSyn protein, which was then dialyzed using a buffer containing 10 mM Tris and 50 mM NaCl (pH 7.5). Endotoxins were removed using a high-capacity endotoxin removal kit (PI88276) and then assessed using an endotoxin quantification kit (A39552). The protein concentration was estimated using absorbance at 280 nm with an extinction coefficient of 7450 M^−1^ cm^−1^. Purified mouse αSyn monomer protein was used to generate αSyn preformed fibrils. Specifically, monomeric αSyn protein was diluted to 5 mg/mL in the buffer (150 mM KCl and 50 mM Tris-HCl), incubated at 37 °C with shaking for 7 days, and centrifuged for 10 min at 13,200 rpm^20^. The protein pellet was re-suspended in half of the initial volume of the solution.

Fibril solution (5 μl) was incubated with 5 μl of 8 M guanidinium chloride at room temperature for one hour, and the concentration of PFFs was measured using absorbance at 280 nm. PFFs were diluted at 5 mg/mL and 22–25 μl aliquots were stored at −80 °C until use. On the day of injection, an aliquot of PFFs (22–25 μl at 5 mg/mL) was thawed at room temperature and sonicated using Qsonica 700 W cup horn sonicator at 30% amplitude using 3 s on/2 s off cycle for 15 min at 15 °C. The size of sonicated PFF (30–70 nm segments) was estimated and confirmed using dynamic light scattering (DynaPro NanoStar from Wyatt technology). Details of the generation and validation of αSyn PFFs can be found here: 10.17504/protocols.io.bhhrj356.

### Stereotaxic brain surgery

Isoflurane (2–3%) was used to induce anesthesia. Mice were placed into a stereotaxic frame (Stoelting, Model: 51730 M) with the head fixed using ear bars. A feedback controller was used to maintain and monitor the body temperature. To induce αSyn pathology in the motor cortex, sonicated PFFs (2 µl at 5 µg/µl) were delivered into the dorsolateral striatum [from bregma in mm, anteroposterior (A-P) = +0.2, mediolateral (M-L) = −2.3, dorsoventral (D-V) = −2.9] through a glass pipette attached to a Nanoliter injector (NANOLITER-2020, World Precision Instrument, FL, USA) with a rate of 0.4 µl per minute. Injection glass pipettes were made by a vertical pipette puller (Model 720, David Kopf Instruments, CA, USA). Mice receiving αSyn monomers or phosphate-buffered saline (PBS) were used as controls. Retrobeads (1:10 dilution, Lumafluor) were mixed with PFFs or monomers or PBS, as appropriate, and co-injected into the dorsolateral striatum or the spinal cord (C7-8) to retrogradely label ITNs or CSNs, respectively. To study thalamocortical transmission using optogenetics, AAV9-hSyn-ChR2(H134R)-eYFP (Addgene#127090, RRID:Addgene_127090, volume = 300 nl, titer = 3.6 × 10^12^ GC/ml) were stereotaxically injected into the motor thalamus centered at the ventromedial subregions (from bregma in mm, A-P = −1.3, M-L = +0.75, D-V = −4.3). Detailed procedures of stereotaxic surgery can be found on Protocols.io (10.17504/protocols.io.rm7vzye28lx1/v1).

### Spinal cord surgery

Mouse spinal cord surgery was performed as described previously^21^. Mouse was placed into a stereotaxic frame (Stoelting) and anesthetized using 2–3% isoflurane. Body temperature was maintained and monitored using a heating pad connected to a feedback controller. An incision (1 cm) was made over the spinal cord, and vertebrae were exposed using retractors. Once the C7 and C8 segments were located, Retrobeads and/or PFFs (1 µl at 5 µg/µl) were injected into the spinal cord (500 µm away from the midline, 700 µm in depth) via a glass pipette mounted on a Nanoliter injector (NANOLITER-2020, WPI, Florida, USA) at a rate of 0.2 µl per minute. Details of mouse spinal cord injections can be found on protocols.io: 10.17504/protocols.io.81wgbz5e3gpk/v1.

### Preparation of brain slices for electrophysiology

Mouse was anesthetized using avertin (300 mg/kg) and perfused transcardially using an ice-cold sucrose-based cutting solution containing 230 mM sucrose, 26 mM NaHCO_3_,10 mM glucose, 10 mM MgSO_4_, 2.5 mM KCl, 1.25 mM, NaH_2_PO_4_, 0.5 mM CaCl_2_, 1 mM sodium pyruvate, and 0.005 mM L-glutathione. The mouse brain was carefully dissected, and coronal brain sections (250 µm in thickness) containing the motor cortex were prepared using a VT1200S vibratome (Leica Microsystems Inc., Deer Park, IL; RRID:SCR_018453). Brain sections were then kept in normal artificial cerebrospinal fluid (aCSF) containing 126 mM NaCl, 26 mM NaHCO_3_,10 mM glucose, 2.5 mM KCl, 2 mM CaCl_2_, 2 mM MgSO_4_, 1.25 mM NaH_2_PO_4_,1 mM sodium pyruvate, and 0.005 mM L-glutathione. Slices were incubated in aCSF at 35 °C for 30 min and then kept at room temperature until recording. Details of brain section preparation can be found on Protocols.io (10.17504/protocols.io.36wgqj2eovk5/v1).

### Ex vivo electrophysiology recording

Brain sections were transferred to a recording chamber perfused with synthetic interstitial fluid (SIF) containing 126 mM NaCl, 26 mM NaHCO_3_,10 mM glucose, 3 mM KCl, 1.6 mM CaCl_2_,1.5 mM MgSO_4_, and 1.25 mM NaH_2_PO_4_ at a rate of 3.5 mL/min. SIF solution was equilibrated with 95% O_2_ and 5% CO_2_ and maintained at 33–34 °C via a feedback-controlled in-line heater (TC-324C, Warner Instruments). Neurons were visualized by a 60x water immersion objective lens (Olympus, Japan) using a SliceScope Pro 6000 system integrated with a charge-coupled device camera (SciCam Pro, Scientifica, UK). Whole-cell patch-clamp recording was performed using a MultiClamp 700B amplifier (Molecular Devices, San Jose, CA; RRID:SCR_018455) and Digidata 1550B controlled by pClamp 11 software (Molecular Devices, San Jose, CA; RRID:SCR_011323). Glass pipettes (BF150-86-10, Sutter Instruments,) were prepared using a micropipette puller (P1000, Sutter Instruments, Novato, CA, USA; RRID:SCR_021042) and filled with one of the following internal solutions: (1) K-gluconate-based internal solution (140 mM K-gluconate, 3.8 mM NaCl, 1 mM MgCl_2_, 10 mM HEPES, 0.1 mM Na_4_-EGTA, 2 mM ATP-Mg, and 0.1 mM GTP-Na pH = 7.3, mOsm = 290); or (2) Celsium-methanesulfonate-based internal solution (120 mM CH_3_O_3_SCs, 2.8 mM NaCl, 10 mM HEPES, 0.4 mM Na_4_-EGTA, 5 mM QX314-HBr, 5 mM phosphocreatine, 0.1 mM spermine, 4 mM ATP-Mg, and 0.4 mM GTP-Na, pH = 7.3, mOsm = 290). Biocytin (0.2%) was included in the K-gluconate internal solution to label the recorded neurons for morphology studies. Ionotropic glutamatergic and GABAergic synaptic transmission was blocked by a cocktail of DNQX (20 μM), D-APV (50 μM), and SR-95531 (10 μM) in the recording solution for intrinsic excitability recording. TTX (1 µM) and 4-AP (100 µM) were routinely included in the recording solution to isolate monosynaptic thalamocortical transmission. In a subset of experiments, the HCN channels were blocked using a selective channel blocker ZD7288 (20 μM, cat#: HB1152, Hellobio). Details of ex vivo electrophysiological recording can be found on Protocols.io (10.17504/protocols.io.eq2ly7m2rlx9/v1).

### Immunofluorescent staining

#### Immunohistology of αSyn pathology and biocytin

After electrophysiology recordings, the brain sections (250 µm) containing biocytin-filled neurons were placed into a 4% paraformaldehyde (PFA) solution overnight at 4 °C. Brain sections were then rinsed three times using phosphate-buffered saline (PBS) and incubated with 2% normal donkey serum in 0.5% Triton-X-100 PBS solution for 1 h, followed by incubation with primary antibody against pS129 αSyn (Rabbit, 1:10,000, Abcam, #1536-1, RRID: AB_562180) overnight at room temperature. The brain sections were rinsed three times with PBS and incubated with the secondary antibody AlexaFluor 488 donkey anti-rabbit IgG (1:500, cat#: 711-545-152, Jackson ImmunoResearch Labs, RRID: AB_2313584) and Cy5-conjugated streptavidin (1:1000, cat#: SA1011, Thermo Fisher Scientific) for two hours at room temperature. After 3x rinse with PBS, brain sections were mounted on slides using a mounting medium (H-1000, Vector Laboratories), and cover slipped.

#### Immunohistology of tyrosine hydroxylase (TH)

The remaining brain tissue from slice preparation for electrophysiology was immersed in 4% PFA solution overnight at 4 °C and re-sectioned (70 µm) using a VT1000s vibratome (Leica Biosystems, Deer Park, IL; RRID:SCR_016495). Slices containing the striatum and the substantia nigra pars compacta were collected for TH or/and phospho-Ser129 (pS129) αSyn immunohistochemistry using the following primary and secondary antibodies: primary antibodies [mouse anti-TH (1:2000, cat#: MAB318, MilliporeSigma; RRID: AB_2201528) and rabbit anti-pS129 α-Syn (1:10,000, Abcam, ab51253, RRID: AB_869973)] and secondary antibodies [Alexa Fluor 488 donkey anti-mouse IgG (1:500, cat#715-545-150; Jackson ImmunoResearch Labs, RRID: AB_2340846) and Alexa Fluor 647 donkey anti-rabbit IgG (1:500, cat#711-605-152, Jackson ImmunoResearch Labs, RRID: AB_2492288)]. Details of immunofluorescent staining can be found on Protocols.io (https://www.protocols.io/view/immunofluorescent-staining-3byl4bq9ovo5/v1).

#### Quantification of αSyn pathology in the motor cortex

The coronal sections of the motor cortex with the following A-P coordinates from the bregma (in mm): +1.94, +1.70, +1.54, and +1.18, were used for quantification of αSyn pathology. The brain sections were manually aligned to the mouse brain atlas of Franklin and Paxinos (5^th^ Edition, 2019, ISBN: 9780128161579) to recognize M1 and M2. The cortical layer boundaries were determined based on a previous report^22^: layer 1 (0–0.1 mm from the pia), layer 2/3 (0.1–0.4 mm), layer 5 A (0.4–0.55 mm), layer 5B (0.55–0.8 mm), and layer 6 (0.8–1.0 mm). ImageJ (NIH, https://imagej.net/, RRID: SCR_003070) was used to quantify the proportion of areas occupied by pS129 αSyn pathology, as reported in our recent work^23,24^.

### Confocal imaging

Immunofluorescent images were collected using a Nikon A1R Confocal Laser Scanning Microscope (RRID:SCR_020317). The pS129-ir αSyn pathology in the cortical area was imaged under a 20x objective lens and quantified in ImageJ (NIH, https://imagej.net/, RRID: SCR_003070). Biocytin-filled cortical neurons were imaged under a 20x lens (NA = 0.75, x/y, 1024/1024 pixels, z-step = 1 µm). Spines of biocytin-filled cortical neurons were imaged under a 100x objective lens (NA = 1.45, x/y, 1024/1024 pixels, z-step = 0.5 µm). Spine density was assessed from 2-3 segments of basal dendrites (20–30 µm in length) at a distance between 50 and 100 µm measured from the soma, which was reconstructed manually using the filament tracer function of Imaris software (Version 10.1.1, Oxford, UK, http://imaris.oxinst.com, RRID: SCR_007370). Details of confocal imaging can be found on Protocols.io: https://www.protocols.io/view/confocal-imaging-and-digital-image-analysis-3byl4jmxzlo5/v1.

### Data analysis and statistics

Electrophysiology data were analyzed using Clampfit software (Version 11.1, Molecular Devices, San Jose, USA, RRID: SCR_011323). The amplitude of EPSCs in response to blue light stimulation was quantified to measure synaptic connection strength. Confocal images were analyzed using Imaris (Version 10.1.1, Oxford, UK, http://imaris.oxinst.com, RRID: SCR_007370) or ImageJ (NIH, https://imagej.net/, RRID: SCR_003070) for spine density quantification or Sholl analysis, respectively. GraphPad Prism (Version 10, GraphPad Software, http://www.graphpad.com, RRID: SCR_002798) was used for statistics analysis. Non-parametric, distribution-independent Mann-Whitney U (MWU) test was used to compare the median of two groups, and the Kruskal-Wallis test was used to compare the median of three or more groups. Two-way analysis of variance (ANOVA) was used to compare the main effects of group differences in the amplitude of EPSCs or the frequency of action potentials across a range of stimulation intensities, followed by Šídák’s multiple comparisons test. *P* < 0.05 was considered as statistically significant.

## Results

### Layer- and cell-type-specific distribution of αSyn pathology in the motor cortex following the intrastriatal PFFs injection

In the intrastriatal PFFs seeding model, αSyn pathology reaches and stays at peak levels in rodent brains at 3 months post-injection (mpi)^25–28^. Thus, all studies in the following sections were conducted at 3 mpi when cortical pathology was robust unless stated otherwise.

**Fig. 1:**
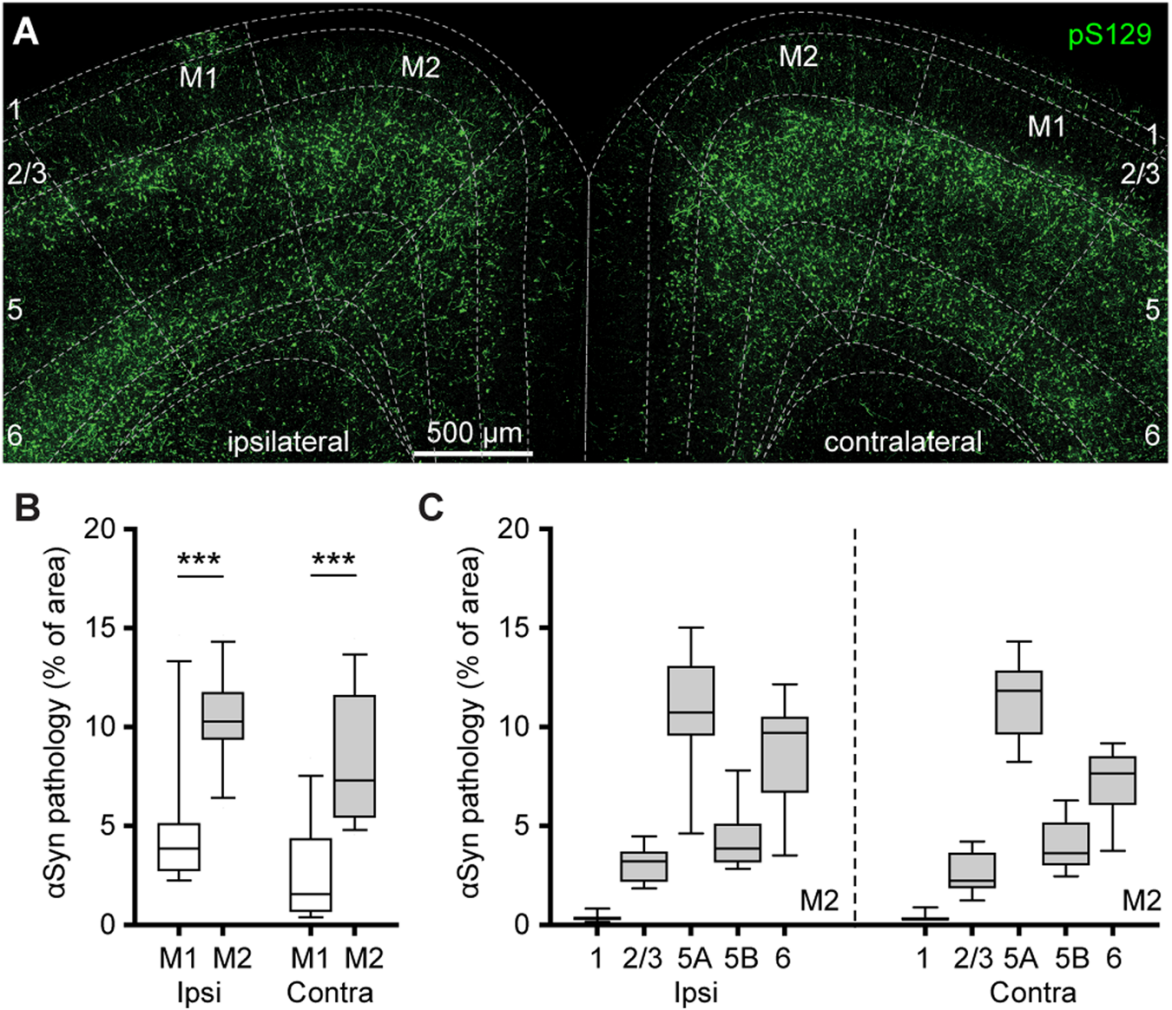
PFFs injection into the dorsal striatum seeds αSyn pathology in the motor cortex. **A** Representative confocal images showing the pS129^+^ αSyn pathology in the motor cortex at 3 months-post-injection (mpi). **B** The proportion of the motor cortical area covered by the pS129^+^ αSyn aggregation. (Ipsilateral, M1 = 3.9 [2.7, 5.2]%, M2 = 10.3 [9.3, 11.8]%, *n* = 12 slices/4 mice for each group; *p* = 0.0005, Mann–Whitney U (MWU) test. Contralateral, M1 = 1.6 [0.6, 4.4]%; M2 = 7.3 [5.4, 11.7]%, *n* = 12 slices/4 mice for each group, *p* = 0.0001, MWU). **C** The proportion of the different cortical layers covered by pS129^+^ αSyn aggregation. In B and C, the αSyn pathology was quantified as the percentage of cortical regions occupied by the pS129 immunoreactivity.

We detected robust phosphorylated αSyn at serine129 (pS129)-immunoreactive (ir) aggregates, indicative of pathological αSyn, in the primary and secondary motor cortices of both hemispheres (M1 and M2, respectively) at 3 mpi (Fig. 1A). No pS129-ir αSyn aggregates were detected in the cerebral cortices of either PBS- or αSyn monomer-injected mice (Supplementary Fig. 1A), as reported previously^26,27,29^. Moreover, the level of αSyn pathology was higher in the M2 than the M1 in both hemispheres (Fig. 1B). In M2, pS129-ir αSyn aggregates were not evenly distributed across the cortical layers; instead, they showed the highest levels in layer 5 A, followed by layer 6, layer 5B, layers 2/3 and 1 (Fig. 1C, see also ref. ^30^).

In PFFs-based models, the development of αSyn pathology in the cortex involves the uptake of PFFs at the corticostriatal axonal terminals^27,31^. Thus, the layer-specific accumulation of αSyn aggregates in M1/M2 is consistent with the anatomical and physiological studies showing that the corticostriatal projection neurons are mainly found in layers 5 in rodent brains^32–34^.

**Fig. 2:**
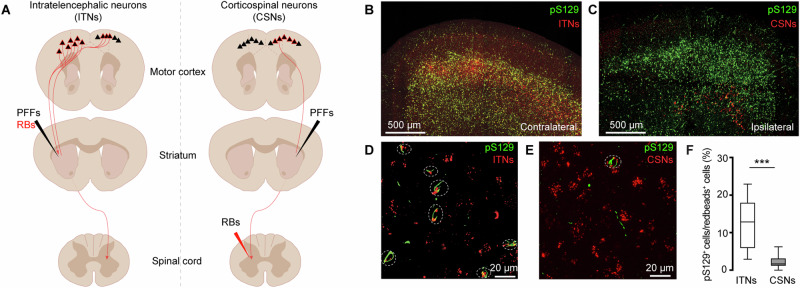
Cell subtype-specific accumulation of αSyn aggregates in the motor cortex. **A** Strategies to retrogradely label subtypes of cortical neurons. Note that intrastriatal PFFs injections labeled both ITNs and CSNs in the ipsilateral motor cortex but only the ITNs in the contralateral motor cortex. Thus, the analyses of ITNs and CSNs were performed from the contralateral and ipsilateral hemispheres, respectively. **B**–**E** Representative images showing the co-localization of Redbeads labeling with pS129^+^ αSyn pathology in motor cortex at low (**B**–**C**) and high (**D**–**E**) magnifications. **F** Summarized results showing that a higher percentage of ITNs than CSNs in M2 were pS129^+^ αSyn positive (ITNs = 12.9 [5.9, 18.0]%, *n* = 11 slices/4 mice; CSNs = 1.7 [1.1, 3.2]%, *n* = 12 slices/4 mice; *p* < 0.0001, MWU).

Since the M2 showed robust pathology, we further interrogated the effects of αSyn aggregation on its microcircuits. The intratelencephalic and corticospinal neurons (a subset of PTNs) are mainly located in layers 5 A and 5B of M2, respectively^34,35^. Considering the striking difference in the pathology levels between layer 5 subregions, we tested the hypothesis that ITNs develop heavier αSyn aggregation than CSNs. To estimate the proportion of aggregates-bearing ITNs and CSNs, we unilaterally injected (1) Retrobeads into either the dorsal striatum to label ITNs or the spinal cord to label CSNs and (2) PFFs into the dorsal striatum for seeding αSyn pathology in the cortex (Fig. 2A). It is known that motor cortical ITNs send bilateral projections to the striatum, and CSNs send collateral projections to the ipsilateral striatum^34^. To quantify the proportion of aggregates-bearing ITNs, we focused on the Retrobeads-labeled neurons in the contralateral M2 relative to the striatum injected with PFFs (Fig. 2B). Similarly, to quantify the proportion of aggregates-bearing CSNs, we focused on the Retrobeads-labeled neurons in the ipsilateral M2 relative to the striatum injected with PFFs (Fig. 2C). Red Retrobeads-labeled ITNs were found broadly in layer 5 of M2 and largely overlapped with pS129-ir αSyn aggregates (Fig. 2B). In contrast, Retrobeads-labeled CSNs were restricted in layer 5B and nearly completely separated from the cortical subregions covered by pS129-ir αSyn aggregates (Fig. 2C). Quantification of the proportion of Retrobeads puncta colocalized with pS129-ir aggregates (Fig. 2D, E) showed that the percentage of pS129-ir ITNs was significantly higher than that of pS129-ir CSNs (Fig. 2F).

**Fig. 3:**
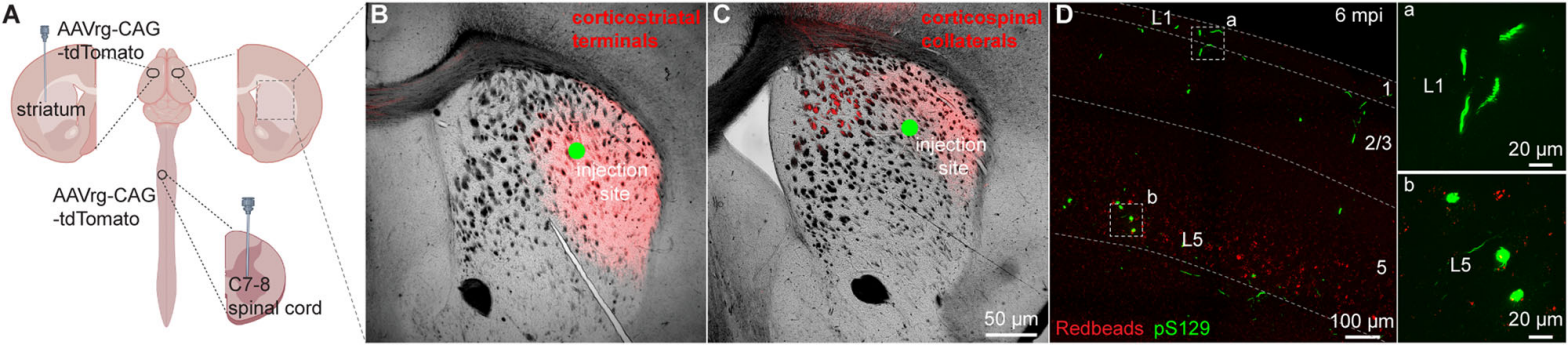
PFFs injection into the spinal cord induces moderate αSyn pathology in the motor cortex. **A** Overall strategies to retrogradely label the ITNs and CSNs and their axonal fields in the striatum. Representative images showing the collateral axonal projections of ITNs (**B**) and CSNs (**C**) in the striatum. The green dots indicate the approximate PFFs injection sites in the dorsolateral striatum. **D** Representative images showing the αSyn pathology in the motor cortex at 6 mpi time point following PFFs injection into the spinal cord. (a, b) Insets showing a high magnification of αSyn pathology from the cortical regions highlighted with the squares in layers 1 (a) and 5 (b).

Given that the seeding of αSyn aggregates depends on the exposure and uptake of PFFs at axon terminals, the lack of αSyn pathology in CSNs was likely due to insufficient or unsuccessful uptake of PFFs by their collaterals in the dorsal striatum. To visualize the corticostriatal axonal field, we unilaterally injected AAVrg-tdTomato into the striatum or the spinal cord (Fig. 3A). As expected, ITNs axonal projections in the striatum had a more diffused pattern of innervation, occupying a larger striatal subregion than the CSNs (Fig. 3B, C). Notably, while the PFFs injection sites stayed within the ITNs axon terminal field (Fig. 3B), they largely avoided the CSNs axon terminal field in the dorsal striatum (Fig. 3C). Differences in the topographic pattern of corticostriatal axonal projections may contribute to the distinct levels of αSyn aggregates between the ITNs and CSNs. To further test this hypothesis, we injected PFFs and Readbeads into the spinal cord (Fig. 3A). At 6 mpi, we found a mild level of αSyn aggregates in M2, including cytoplasmic aggreges in layer 5B and fibril-like aggregates in layer 1 that were supposed to be pathology-bearing apical dendrites of CSNs (Fig. 3D).

Together, these results suggest that αSyn aggregates can be induced in both ITNs and CSNs of M2, but the extent of pathology seems to be greater in ITNs than in CSNs, perhaps depending on where the seeding process starts.

### M2 neuronal hyperexcitability associated with αSyn aggregation

**Fig. 4:**
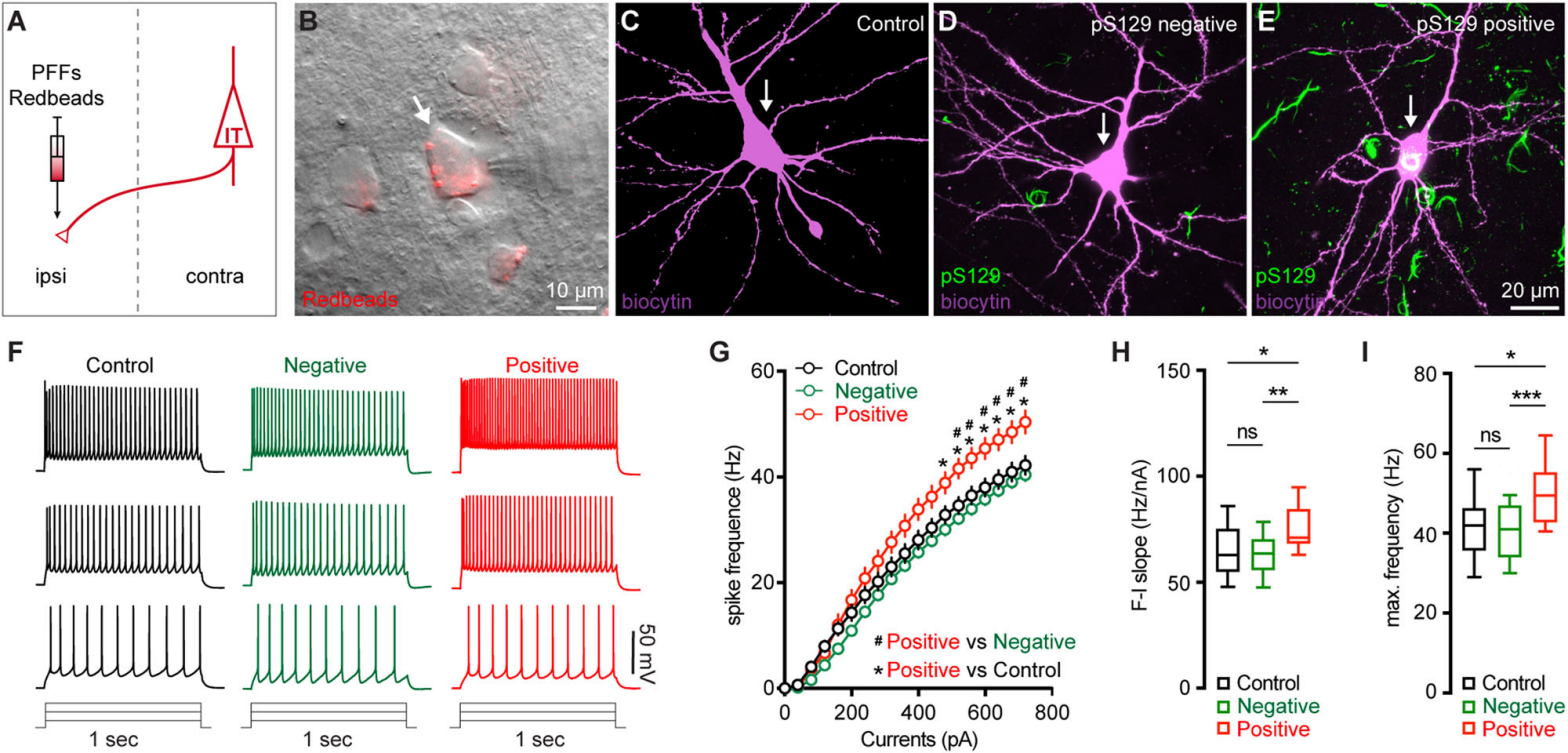
M2 neuronal hyperexcitability associated with αSyn pathology. **A** Diagram showing the experimental design for co-injection of αSyn PFFs with Retrobeads into the contralateral dorsal striatum. **B** Representative images showing a Retrobeads labeled ITN in layer 5 of M2 that was targeted for electrophysiology recording and intracellular dialysis of biocytin.Representative images showing biocytin labeled M2 ITNs neurons that were from controls (**C**), or pS129-negative (**D**) and pS129-positive (green, **E**) from PFFs-injected mice. **F** Representative spike trains of ITNs evoked by somatic current injections (+160 pA, +320 pA, and +480 pA for 1 s) from controls (left), as well as pS129-negative (middle) and pS129-positive (right) cells. **G** The frequency-current (F-I) relationship of controls as well as pS129-negative and positive cells. Controls = 34 neuron/5 mice, pS129-negative = 53 neurons/9 mice, pS129-positive = 14 neurons/9 mice. *p* < 0.0001, pS129-positive group versus controls or pS129-negative ones, mixed effects model followed by Sidak’s test. **H, I** Summarized results showing increased F-I slopes (H, Controls = 34 neurons/5 mice; pS129-negative = 53 neurons/9 mice; pS129-positive = 14 neurons/9 mice; *p* = 0.0068, Kruskal-Wallis test followed by Dunn’s test) and increased maximum frequency of firing in pS129-positive group relative to controls or pS129-negative ones (I, Control = 34 neurons/5 mice; pS129-negative = 53 neurons/9 mice; pS129-positive = 14 neurons/9 mice; *p* = 0.0039, Kruskal-Wallis test followed by Dunn’s test).

Next, we sought to understand the functional consequences of αSyn aggregation on the physiology and morphology of M2 neurons. Given their preferential accumulation of αSyn aggregates, we performed whole-cell patch-clamp recordings from retrogradely labeled M2 ITNs from the contralateral hemisphere to the PFFs-injected striatum (Fig. 4A). We labeled all recorded neurons using biocytin through the recording pipettes for *post hoc* immunohistochemical examination of pS129-ir αSyn pathology (Fig. 4B–E). None of the biocytin-labeled M2 neurons from PBS- or monomer-injected mice showed pS129-ir αSyn pathology (Fig. 4C). In PFFs-injected mice, we detected somatic αSyn pathology in 14 out of 67 retrogradely labeled ITNs (i.e., pS129-positive hereafter, Fig. 4E), but not in the other 53 cells (i.e., pS129-negative hereafter, Fig. 4D). The small proportion of biocytin-labeled ITNs bearing αSyn aggregates is consistent with our initial observations from the histological studies (Fig. 2F).

After the blockade of ionotropic glutamatergic and GABAergic synaptic transmission, we injected a family of currents (1 s) ranging from 0 to 720 pA via the patch pipettes to assess the intrinsic excitability of ITNs. We compared the intrinsic excitability of ITNs of M2 from PBS- and αSyn monomer-injected mice and found no difference between the two groups (Supplementary Fig. 1B–E). Thus, we pooled cells from PBS- and monomer-injected mice as “controls” hereafter. We found that, in response to a given intensity of current injection, pS129-positive ITNs discharged more action potentials (APs) relative to pS129-negative ones in PFFs-injected mice or controls (Fig. 4F, G). In contrast, there was no difference in the overall excitability of pS129-negative ITNs in PFFs-injected mice relative to controls (Fig. 4F, G). We then quantified the frequency-current (F-I) curves of ITNs and found that pS129-positive ITNs exhibited a steeper F-I slop and increased maximal frequency of firing at 720 pA, relative to pS129-negative ITNs from PFFs-injected mice or controls (Fig. 4H, I). Together the above results suggest that the formation of cytoplasmic αSyn aggregates enhances the cellular excitability of ITNs in layer 5 of M2.

### Ionic mechanisms of M2 neuronal hyperexcitability associated with αSyn aggregation

**Fig. 5:**
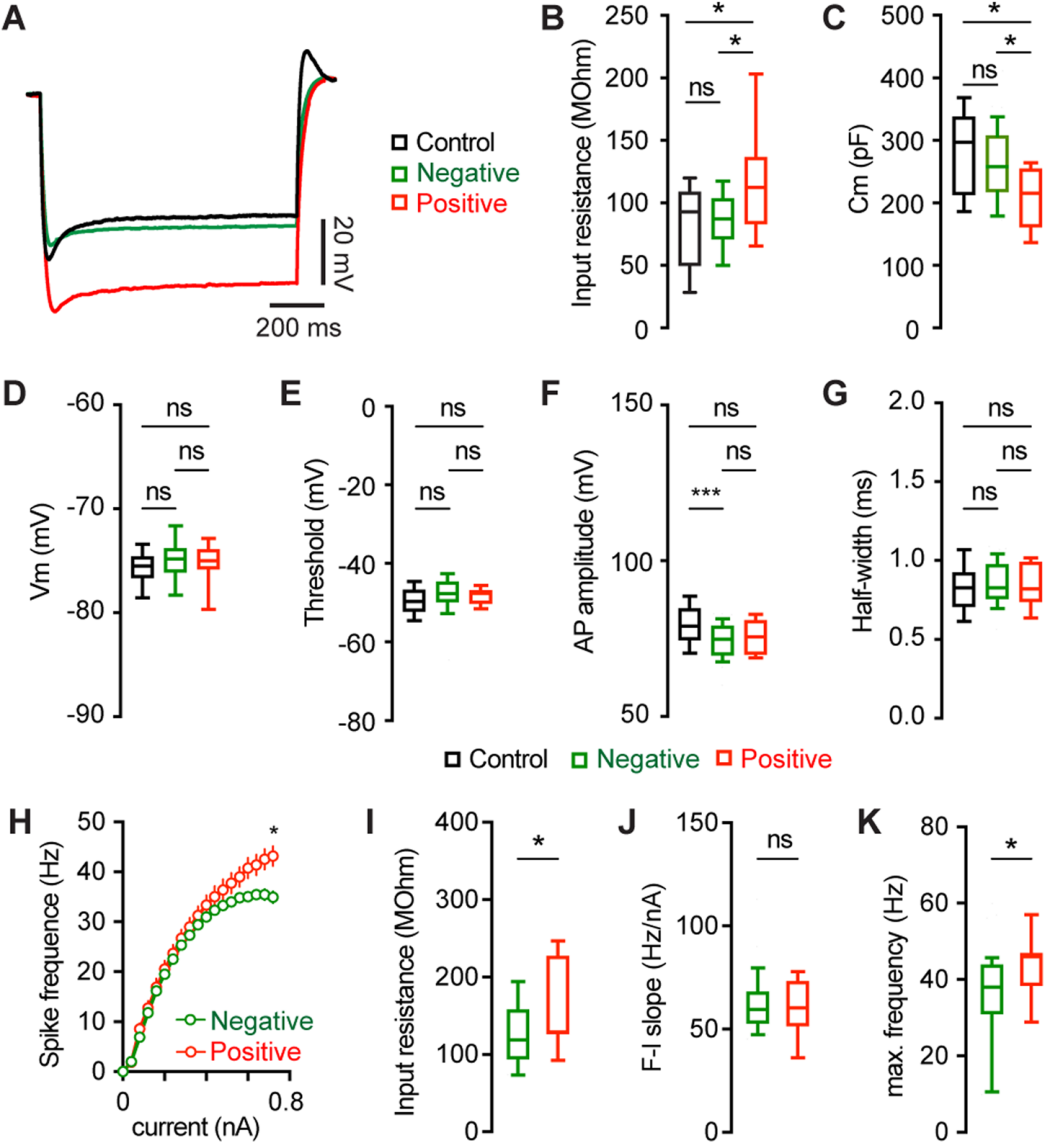
HCN channel dysfunction contributes to the αSyn aggregation-induced hyperexcitability of the M2 ITNs. **A** Representative membrane responses evoked by negative current injections (−240 pA for 1 s) of ITNs from controls and those positive and negative for pS129 aggregation.Box plots showing an increased input resistance (**B**, Control = 93.1 [50.0, 109.0] MOhm, *n* = 34 neurons/5 mice; pS129-negative = 87.4 [71.2, 103.6] MOhm, *n* = 51 neurons/9 mice; pS129-positive = 112.7 [83.4, 136.5] MOhm, *n* = 14 neurons/9 mice; *p* = 0.0293) and a decreased cell capacitance (**C**, control = 297.2 [213.3, 337.9] pF, *n* = 34 neurons/5 mice; pS129-negative = 262.6 [224.5, 308.7] pF, *n* = 52 neurons/9 mice; pS129-positive = 215.4 [161.4, 254.3] pF, *n* = 14 neurons/9 mice; *p* = 0.0025) of ITNs from pS129-positive group relative to control and pS129-negative groups. Box plots showing no or subtle change in the resting membrane potential (**D**, control = −75.5 [−76.6, −74.6] mV, 34 neurons/5 mice; pS129-negative = −74.8 [−76.1, −74.6] mV, *n* = 50 neurons/9 mice; pS129-positive = −75.0 [−76.45, −72.6] mV, *n* = 13 neurons/9 mice; *p* = 0.2287), threshold (**E**, control = −49.7 [−52.3, −46.7] mV, 33 neurons/5 mice; pS129-negative = −47.7 [−49.7, −44.7] mV, 52 neurons/9 mice; pS129-positive = −47.5 [−49.8, −46.8] mV, 14 neurons/9 mice; *p* = 0.0566), AP amplitude (**F**, control = 79.07 [74.63, 84.74] mV, 34 neurons/5 mice; pS129-negative = 74.86 [69.77, 79.05] mV, 52 neurons/9 mice; pS129-positive = 75.61 [70.01, 80.82], 14 neurons/9 mice; *p* = 0.0048), and AP half-width (**G**, control = 0.85 [0.78, 0.92] ms, *n* = 34 neurons/52 mice; pS129-negative = 0.83 [0.76, 0.98], *n* = 52 neurons/9 mice; pS129-positive = 0.82 [0.74, 0.99], *n* = 14 neurons/9 mice; *p* = 0.9943) of ITNs from pS129-positive group relative to controls and pS129-negative groups. Kruskal-Wallis test followed by Dunn’s test. **H**. The F-I relationship of the pS129-positive (9 neurons/4 mice) and -negative (60 neurons/4 mice) ITNs in the presence of HCN channel blocker. *p < 0.05, two-way ANOVA followed by Šídák’s multiple comparisons test. **I, K** Boxplots showing differences in the input resistance (I, pS129-positive = 129.0 [126.2, 227.5] MOhm, *n* = 9 neurons/4 mice; pS129-negative = 119.0 [94.08, 158.5] MOhm, *n* = 60 neurons/4 mice, *p* = 0.04, Mann-Whitney U test), the slope of the F-I curves (**J**, pS129-positive = 60.40 [51.50, 73.30] Hz/nA, *n* = 9 neurons/4 mice; pS129-negative = 59.60 [52.83, 68.30] Hz/nA, *n* = 60 neurons/4 mice, *p* = 0.8, Mann-Whitney U test), and the maximum frequency of firing in pS129-positive relative to pS129-negative ones (K, pS129-positive = 46.0 [38.50, 47.0] Hz, *n* = 9 neurons/4 mice; pS129-negative = 38.0 [31.0, 44.0] Hz, *n* = 61 neurons/4 mice; *p* = 0.025, MWU test).

To understand the ionic mechanisms underlying the hyperexcitability of M2 ITNs, we injected a family of negative current steps to assess their passive membrane properties. Negative current injections evoked larger membrane responses in pS129-positive ITNs than in pS129-negative ones or those from controls (Fig. 5A). Quantitative analyses showed that pS129-positive ITNs exhibited higher input resistance and smaller cell capacitance relative to pS129-negative ITNs from PFFs-injected mice and those from controls (Fig. 5B, C). However, we did not detect changes in other passive membrane properties, including the resting membrane potential (Vm, Fig. 5D), as well as the threshold, amplitude, and half-width of APs (Fig. 5E, F, G).

The hyperpolarization-activated cyclic nucleotide-gated (HCN) channels play a critical role in regulating membrane input resistance and cellular excitability of cortical and hippocampal pyramidal neurons^36,37^. Thus, we tested the hypothesis that HCN channel dysfunction underlies the hyperexcitability of the M2 ITNs associated with pS129 α-Syn aggregation. In the presence of a selective HCN channel blocker ZD7288 (20 μM), we unbiasedly sampled and assessed the intrinsic excitability of ITNs from PFFs-injected mice followed by *post hoc* IHC examination of the cytoplasmic pS129 aggregates. This unbiased strategy allowed us to cluster ITNs based on the presence and absence of pathology and compare their intrinsic excitability. Bath application of ZD7288 (20 μM) abolished the sag potentials in ITNs upon hyperpolarizing current injections, indicating an effective pharmacological blockade of HCN channels. In the presence of ZD7288, the hyperexcitability of the pS129-positive ITNs was abolished at small to moderate intensities of current injections but remained at larger depolarizing current injections (Fig. 5H, K). Moreover, we also detected a moderate and significant increase in the input resistance of pS129-positive ITNs relative to pS129-negative ones (Fig. 5I). There was no difference in the slope of F-I curves between the two categories of ITNs (Fig. 5J). Collectively, the above data suggest that the hyperexcitability of M2 ITNs is partially caused by downregulated HCN channel function.

### Morphological changes of M2 neurons associated with αSyn aggregation

**Fig. 6:**
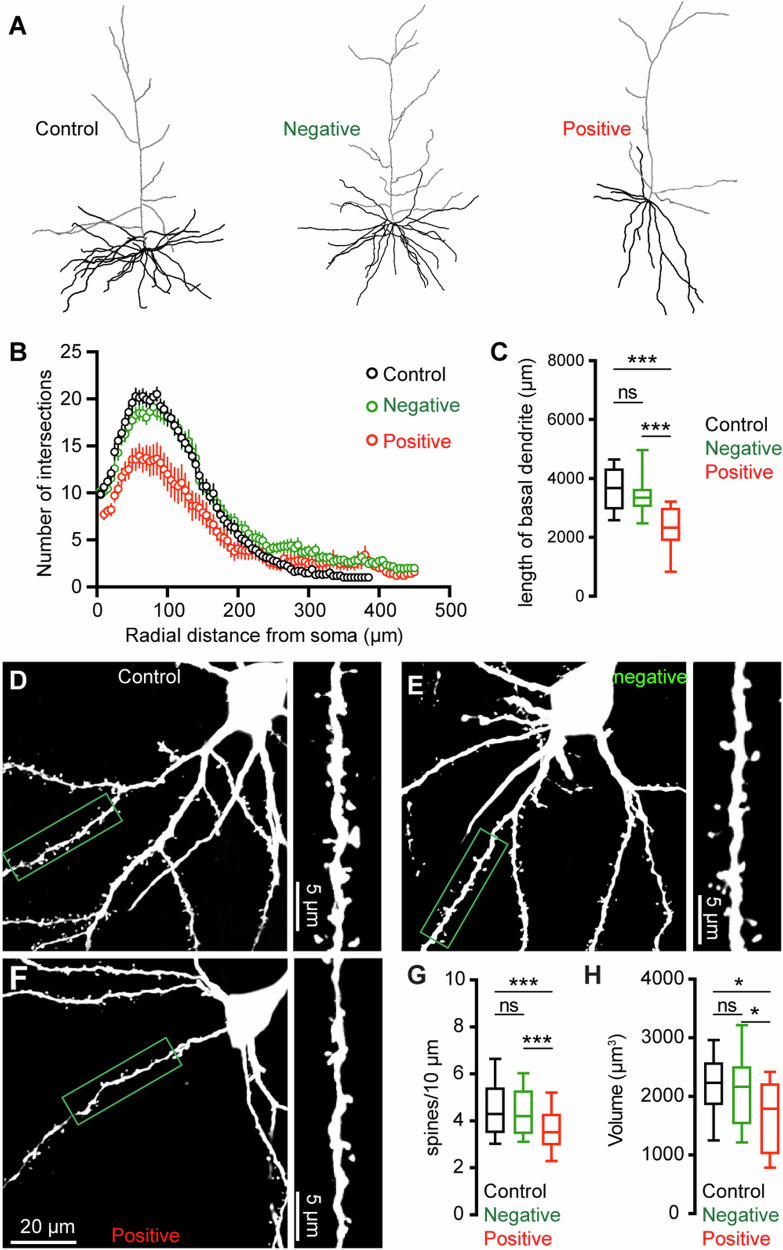
αSyn pathology induces morphological changes in ITNs. **A** Representative reconstructed ITNs from control, pS129-negative, and pS129-positive groups. Black and gray traces indicate basal and apical dendrites, respectively. **B** Sholl analysis showing a reduced dendritic branching (*p* < 0.0001, mixed effects model followed by Sidak’s test). **C** Box plots showing decreased total length of the basal dendrites (control = 3679 [2951, 4328] μm, *n* = 22 neurons/4 mice; pS129-negative = 3354 [3050, 3641] μm, 25 neurons/6 mice; and pS129-positive = 2330 [1886, 2998] μm, 14 neurons/9 mice, *p* < 0.0001) of pS129-positive ITNs relative to control and pS129-negative ones. Representative images of biocytin-filled ITNs and segments of dendritic spines from control (**D**), pS129-negative (**E**), and pS129-positive (**F**) groups. **G** Box plots showing a decreased spine density in pS129-positive ITNs relative to controls and pS129-negative ones (spine density, controls = 4.290 [3.488, 5.401]/10 μm, *n* = 66 segments/4 mice; pS129-negative = 4.202 [3.460, 5.285]/10 μm, *n* = 75 segments/6 mice; pS129-positive = 3.521 [2.979, 4.297]/10 μm, *n* = 41 segments/9 mice; *p* = 0.0056. **H** Boxplots showing decreased soma volume of pS129-positive ITNs relative to controls and pS129-negative ones. (Control = 2234 [1860, 2579] μm^3^, *n* = 31 neurons/5 mice; pS129-negative = 2169 [1536, 2514] μm^3^, *n* = 32 neurons/9 mice; pS129-positive = 1794 [1020 to 2225] μm^3^, *n* = 14 neurons/9 mice; *p* = 0.0331, pS129-positive versus controls; *p* = 0.0359, pS129-positive versus pS129-negative. Kruskal-Wallis test followed by Dunn’s test for **C**, **G**, **H**.

To further understand the impact of αSyn aggregation on cortical microcircuits, we filled Retrobeads-labeled M2 ITNs with biocytin via the patch pipettes to study potential changes in their morphology. Biocytin-labeled dendritic tree and cell body were visualized by a confocal microscope, followed by a three-dimensional reconstruction for analysis. Somatic pS129-ir αSyn aggregates were immunohistochemically examined for all M2 ITNs from PFFs-injected mice, which were then analyzed and presented separately. We found a significant reduction of dendritic arborization of pS129-positive ITNs in M2 relative to pS129-negative ITNs from PFFs-injected mice or those from controls (Fig. 6A). Sholl analysis showed significantly fewer dendritic branches within 200 μm from the soma of pS129-positive ITNs relative to pS129-negative ITNs from PFFs-injected mice or controls (Fig. 6B). A large portion of proximal dendritic branches was from the basal dendrites in layer 5. Consistently, we found that pS129-positive ITNs had a shorter length of basal dendrites compared to pS129-negative ITNs from PFFs-injected mice or controls (Fig. 6C). Moreover, there was a significant loss of spines on the basal dendrites of pS129-positive ITNs, relative to pS129-negative ITNs from PFFs-injected mice or controls (Fig. 6D–G). Last, we also conducted a three-dimensional reconstruction of M2 ITNs and quantified their soma volume to assess the size of the cell bodies. We found a significant reduction in the soma size of pS129-positive ITNs of M2 relative to pS129-negative ones from PFFs-injected mice or controls (Fig. 6H), suggesting a shrinkage of ITNs bearing αSyn aggregates, which is consistent with the decreased cell capacitance (Fig. 5C).

### αSyn aggregation does not alter motor thalamic inputs to ITNs of M2

**Fig. 7:**
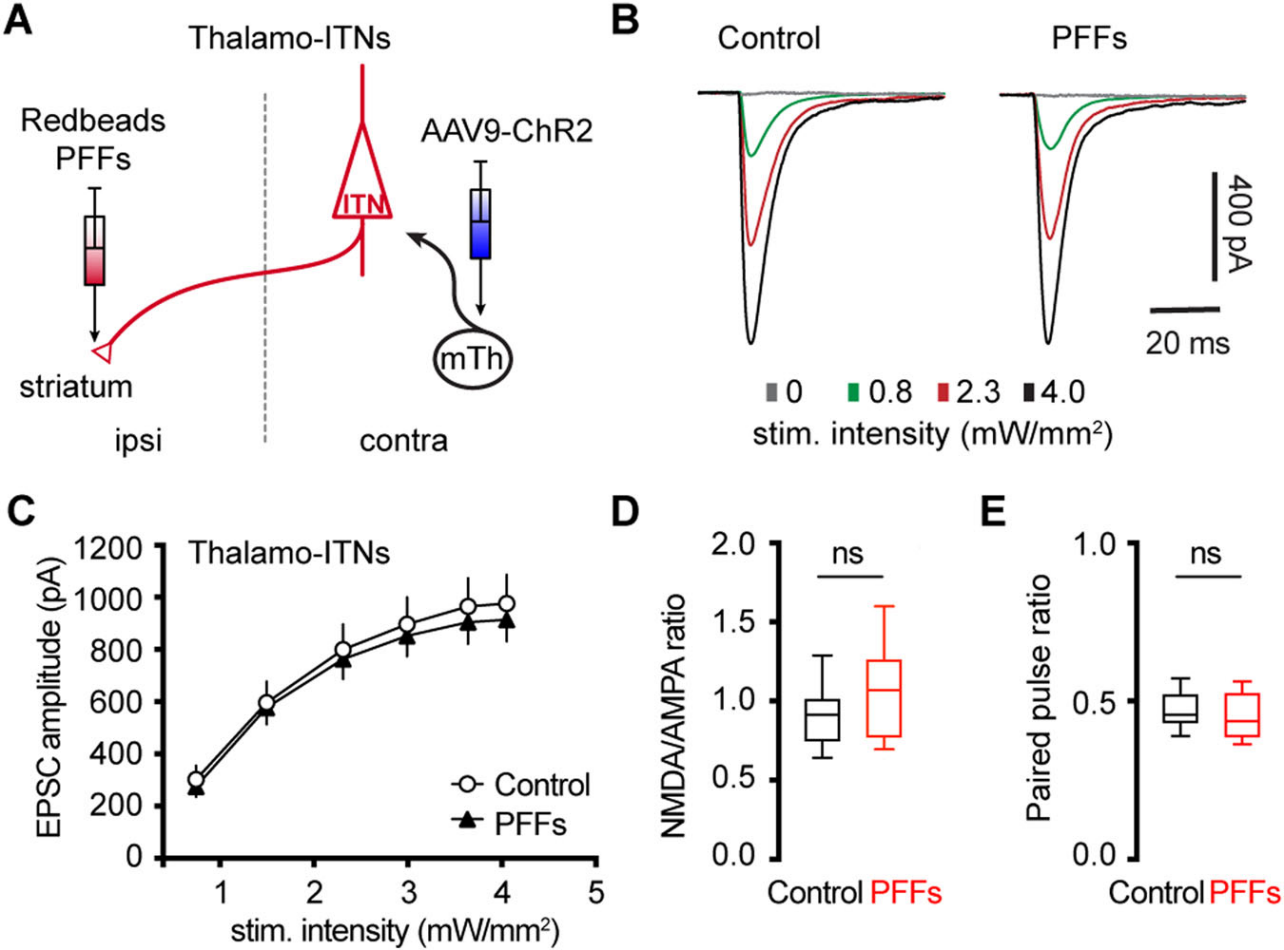
No change in the thalamocortical transmission to M2 ITNs in the PFFs seeding model. **A** Diagram showing the experiment design to study thalamocortical transmission of M2 ITNs. Representative traces of optogenetically evoked EPSCs in ITNs across different stimulation intensities from control and PFF groups (**B**) and the summarized results (**C**, control = 26 neurons/5 mice; PFF = 18 neurons/3 mice. *p* = 0.7391, mixed effects model followed by Sidak’s tests). **D** Box plots showing no change in the NMDA/AMPA ratio at thalamocortical synapses to M2 ITNs between controls and PFFs-injected mice (control = 0.91 [0.75, 1.02], 26 neurons/5 mice; PFFs = 1.07 [0.77, 1.26], 18 neurons/3 mice, *p* = 0.09, MWU). **E** Box plots showing no change in the paired pulse ratio at thalamocortical synapses to M2 ITNs between controls and PFFs-injected mice (control = 0.46 [0.43, 0.53], 21 neurons/5 mice; PFFs = 0.44 [0.39, 0.53], 16 neurons/3 mice, *p* = 0.44, MWU test).

Glutamatergic motor thalamic inputs to cortical pyramidal neurons form synapses at basal dendrites in layer 5 and distal apical dendrites in layer 1^8,10,38,39^. The significant loss of basal dendritic spines indicates that the thalamic inputs to ITNs may be altered by αSyn aggregation^24^. In the intrastriatal PFFs seeding model, we mixed PFFs with Retrobeads and then injected the mixed solution unilaterally into the dorsal striatum for retrograde labeling of M2 ITNs and seeding of αSyn aggregation (Fig. 7A). The ITNs in the contralateral hemisphere, with intact nigrostriatal and mesocortical dopamine projections, were targeted and studied. Thus, the effect of αSyn aggregation was not confounded by the potential impact of dopamine depletion^8^.

To interrogate the synaptic strength of thalamic inputs to the ITNs of M2, we injected AAV9-ChR2(H134R)-eYFP into the motor thalamus that was contralateral to the hemisphere receiving PFFs/Retrobdeas injections (Fig. 7A). Upon stimulation of ChR2-expressing thalamic axon terminals in M2 by delivering 478 nm light, robust excitatory postsynaptic currents (EPSCs) were recorded using whole-cell voltage-clamp recording at −80 mV, which could be completely abolished by AMPA receptor antagonist DNQX (20 μM). A range of intensities of blue light was delivered to assess the connection strength of thalamo-ITNs synapses in controls and PFFs-injected mice. We found no change in the amplitude of thalamic EPSCs in ITNs from PFFs-injected mice relative to those from controls across a range of light intensities (Fig. 7B, C). Pathological αSyn aggregation may interact with postsynaptic NMDA receptor subunits, as reported in the striatum^40^. To test this possibility in the cortical circuits, we quantified the ratio of NMDA- and AMPA-receptor mediated EPSCs (NMDA/AMPA ratio) at thalamo-ITNs, and found no difference in the NMDA/AMPA ratio between controls and PFFs-injected mice (Fig. 7D). Furthermore, we did not detect a change in paired-pulse ratios (PPR) at the thalamo-ITNs between PFFs-injected mice and controls (Fig. 7E), indicating a lack of alterations of the initial presynaptic release probability following αSyn pathology formation. These data were supported by an absence of prominent αSyn pathology accumulation in the ventromedial region of the motor thalamus (Supplementary Fig. 2). The above results are consistent with recent studies showing low levels of *SNCA* gene expression in the motor thalamus and αSyn protein expression in vGluT2-expressing thalamic neurons and their axon terminals in the cerebral cortex^23,41^.

### Partial dopamine depletion does not alter the excitability and synaptic excitation of corticospinal neurons in M2

**Fig. 8:**
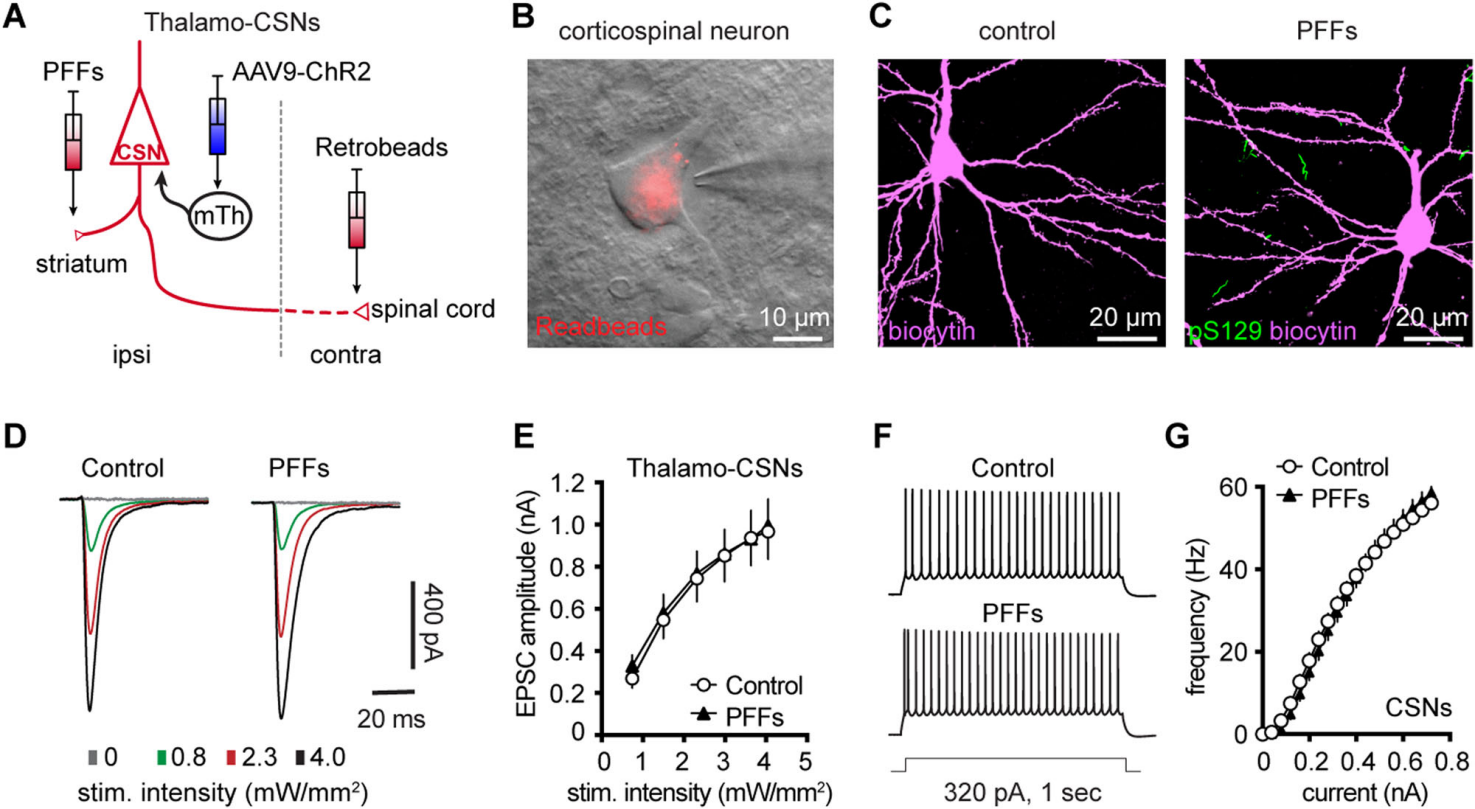
Intrinsic and synaptic properties of M2 CSNs were not affected in the intrastriatal PFFs-seeding model of synucleinopathy. **A** Experimental design to study intrinsic and synaptic properties of corticospinal neurons in the intrastriatal PFFs-seeding model using optogenetic approaches. **B** Representative image of a Retrobeads labeled corticospinal neuron that was targeted for physiological recording and dialysis of biocytin. **C** Representative confocal images showing biocytin-filled corticospinal neurons from controls and PFFs-injected mice. Both neurons were negative of pS129 immunoreactivity in post hoc IHC staining. **D** Representative traces of optogenetically evoked EPSCs in CSNs across different stimulation intensities from controls and PFFs-injected mice. **E** Summarized results show no change in the amplitude of thalamocortical EPSCs in CSNs between controls and PFFs-injected mice (controls = 24 neurons/5 mice; PFF = 24 neurons/4 mice. *P* = 0.8605, mixed effects model followed by Sidak’s tests). **F** Representative AP spike trains of CSNs from controls and PFFs-injected mice in response to somatic current injections (320 pA for 1 sec). **G** Summarized graph showing no changes in the excitability of CSNs from controls and PFFs-injected mice (*p* = 0.8110, Kruskal-Wallis test. control = 28 neurons/5 mice; PFF = 20 neurons/3 mice).

At 3 months post injections, moderate levels of pS129 αSyn pathology accumulated in the ventral tier of the substantia nigra compacta of PFFs-injected mice, but no obvious cell loss was detected (Supplementary Fig. 3A, B). Consistently, the density of TH immunoreactive axon terminals in the ipsilateral striatum decreased by ~30% in PFFs-injected mice (Supplementary Fig. 3E). We recently reported impaired intrinsic excitability and reduced thalamic excitation to motor cortical PTNs in parkinsonian mice with nearly complete dopamine depletion^8,11^. Therefore, although corticospinal neurons did not develop detectable αSyn aggregation in the intrastriatal PFF model (Figs. 2 and 8C), their intrinsic and synaptic properties may be affected by the partial striatal dopamine loss and the associated basal ganglia dysfunction^42,43^. To test this hypothesis, we injected (1) αSyn PFFs into the dorsal striatum to induce the formation of αSyn aggregation in the SNc dopaminergic neurons and partial striatal dopamine depletion (Supplementary Fig. 3B–E); (2) AAV9-ChR2(H134R)-eYFP into the motor thalamus of the same hemisphere as the striatum receiving PFFs injections; and (3) Retrobeads to the spinal cord to retrogradely label corticospinal neurons for electrophysiology recordings (see experiment design in Fig. 8A). Upon optogenetic stimulation of ChR2-expressing thalamic axon terminals in M2, we detected robust thalamic EPSCs in corticospinal neurons, as previously reported^8,38,39^. At 3 mpi, there was no difference in the amplitudes of thalamic EPSCs in corticospinal neurons between controls and the PFFs-injected mice (Fig. 8D, E), suggesting that there was no change in the connection strength of thalamocortical inputs to the corticospinal neurons. Thus, we concluded that a partial loss of striatal dopamine does not affect the connection strength of thalamic inputs to the corticospinal neurons.

To determine if a partial loss of DA can alter the excitability of corticospinal neurons^8,11^, we assessed the intrinsic excitability of M2 CSNs by performing whole-cell current-clamp recordings from PFFs-injected mice and controls at 3 mpi. We found that the CSNs discharged a similar number of APs in response to a range of current injections between groups (Fig. 8F, G). These results suggest that the intrinsic excitability of M2 corticospinal neurons was not altered by the partial dopamine depletion in the intrastriatal PFFs model.

## Discussion

**Fig. 9:**
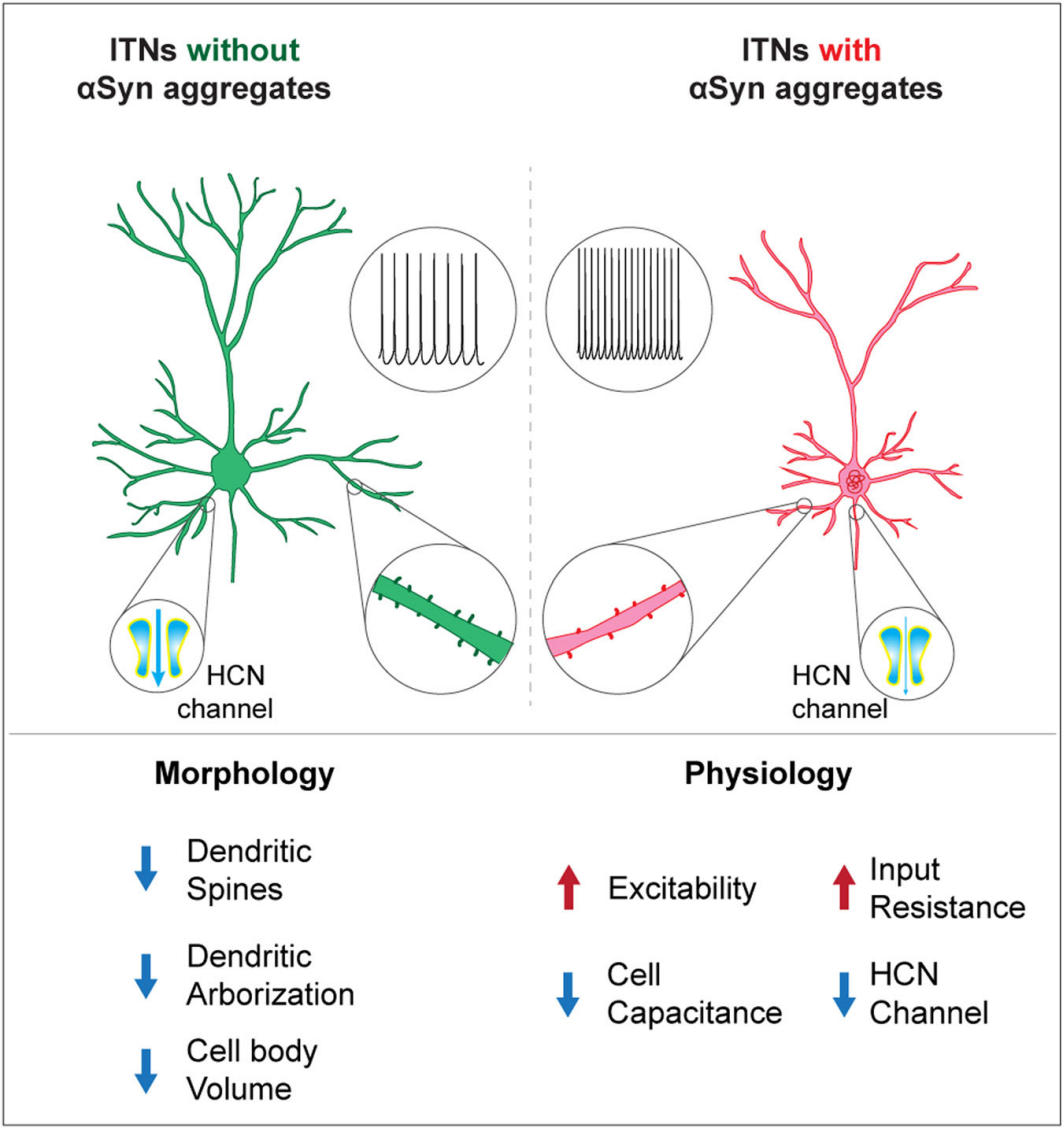
A graph summarizing the morpho-electrophysiological changes of cortical neurons associated with αSyn pathology. The formation of cytoplasmic αSyn aggregates changes the morphological and physiological properties of cortical pyramidal neurons via cell-autonomous mechanisms, involving a downregulated HCN channel function.

The present study demonstrated that (1) the formation of intracellular αSyn aggregates increases the cellular excitability of M2 pyramidal neurons, (2) the increased intrinsic excitability occurs through cell-autonomous mechanisms, and (3) partial degeneration of the nigrostriatal pathway is not sufficient to trigger adaptative changes of the intrinsic and synaptic properties of M2 corticospinal neurons (Fig. 9). Taken together, the present study provides novel insights into cortical pathophysiology in parkinsonism and highlights an unmet need to study the functional impact of αSyn aggregation on cellular and synaptic properties prior to overt cell death to design effective treatment for PD and other synucleinopathies.

Post-mortem analyses of the brains of PD patients and animal models have identified a group of brain regions that show particular vulnerability to accumulation of αSyn aggregations, including the SNc, the locus coeruleus, and the dorsal vagal nucleus, among many others^18^. Compelling evidence suggests that αSyn pathology propagates through brain networks, driving neuronal death and loss of synaptic connections within and between the vulnerable brain regions and cell subtypes^44–47^. However, not all cells bearing αSyn pathology degenerate in the brain, indicating not only a poor correlation between Lewy pathology and cell death but also critical roles of cell-intrinsic mechanisms in gating neurodegeneration^44^. In addition, detrimental consequences of αSyn aggregation to neuronal and synaptic function could be the major driver of phenotype manifestation in PD and other synucleinopathies^48^. Considering the preferential presynaptic location of αSyn, a large body of studies have focused on studying the effects of its abnormal aggregation on synaptic structure and function^31,49,50^. These reports highlight disrupted synaptic transmission at dopaminergic and glutamatergic systems that may underlie either motor or nonmotor deficits associated with αSyn aggregation^24,40,51–54^.

On the other hand, emerging evidence suggests that abnormal aggregation of αSyn triggers adaptive changes in the physiological properties of dopaminergic neurons in the SNc and cholinergic neurons in the vagal nucleus^55–58^. Our studies further expand these observations to the motor cortical glutamatergic circuits, which exhibit Lewy pathology at late stages in PD. A key finding of our study is that αSyn aggregates-bearing cortical pyramidal neurons showed increased excitability relative to those without cytoplasmic αSyn aggregates from the PFFs-injected mice or controls (Fig. 4). Since the intrinsic excitability was assessed in the presence of blockers of the ionotropic glutamatergic and GABAergic receptors, the hyperexcitability of pathology-bearing cortical neurons was likely to be mediated by cell-autonomous mechanisms. These data provide a mechanistic understanding of cortical hyperexcitability observed in vivo, which was hypothesized to be due to a disrupted balance between synaptic excitation/inhibition^59,60^. Mechanistically, we found that the hyperexcitability of M2 pyramidal neurons was associated with increased input resistance and decreased cell capacitance (Fig. 5). Moreover, we documented that pharmacological blockade of the HCN channels largely canceled the hyperexcitability of ITNs (Fig. 5), indicating that downregulated HCN channel function partially contributed to the increased input resistance and cellular hyperexcitability associated with αSyn aggregation. It is worth noting that the pS129-positive ITNs discharged more action potentials in response to large somatic current injections. This indicates the involvement of undefined ionic mechanisms active at robust membrane depolarization in the observed cortical hyperexcitability. These ionic mechanisms may include the abnormal membrane expression and re-distribution of ion channels (e.g., voltage-gated Ca^2+^ and K^+^ channels) as reported in the SNc dopaminergic neurons and vagal cholinergic neurons^55–57,61^. Congruous with physiological changes, pS129-positive neurons showed significant changes in their morphology, including shorter dendrites, dendritic spine loss, and the shrinkage of cell bodies (Fig. 6). These are similar morphological changes as in other cell types across brain regions^24,57,58^. The changes in cell capacitance and morphological features suggest that αSyn aggregates-bearing ITNs are compact and more excitable. The combined physiological and morphological changes of ITNs are consistent with earlier studies showing that dendritic complexity and ion channel expression are morphological determinants of cellular and synaptic excitability^37,62^.

In addition to cell-autonomous adaptations, we cannot exclude the potential contribution of non-cell autonomous mechanisms to neuronal and network hyperexcitability in vivo, such as the involvement of glial activation and neuroinflammatory responses^63,64^.

It has been reported that acute dialysis of neocortical pyramidal neurons with αSyn oligomers reduced their intrinsic excitability, decreased input resistance, and increased cell capacitance, which might be mediated by immediate direct interaction between the αSyn oligomers and the cell membranes^65^. The discrepant results between previous work and the present study are likely due to various technical details, including the structure, concentration, and route of inoculation of the αSyn strains, as well as the subsequent cell biological processes engaged by αSyn oligomer versus Lewy body-like aggregates at distinct time scale^66^.

Though M2 ITNs showed intrinsic hyperexcitability, it does not necessarily mean their output to the striatum and other subcortical regions is enhanced. Contrarily, evidence in the literature suggests a significant reduction of cortical glutamatergic outputs in the model of synucleinopathies that may occur at a relatively earlier time point^23,24,67,68^.

Surprisingly, the loss of spines of the basal dendrites was not associated with alterations in thalamocortical connection strength of ITNs and presynaptic release probability in the M2 (Fig. 7). Given the detrimental effects of αSyn aggregation on presynaptic transmission^24,31,69^, this observation is supported by the absence of pathological αSyn aggregates in the ventromedial thalamus, which is correlated with the relatively low levels of endogenous αSyn in most thalamic subregions^23,70^. However, a limitation of the present study was the lack of distinguishing ITNs based on the absence and presence of αSyn aggregates, which might result in underestimated postsynaptic effects of αSyn aggregation to thalamocortical synaptic connection. Particularly, shortened dendrites and spine loss in ITNs are likely associated with changes in dendritic expression of voltage-gated ion channels, disrupting the nonlinear properties of dendrites. Further studies using advanced circuit interrogation tools with high spatial resolution or computational simulation are warranted on the potential impact of morphological alterations in cortical ITNs on dendritic excitability and synaptic efficacy^62^.

Motor cortical PTNs are preferentially and severely affected by the midbrain dopaminergic neurodegeneration in parkinsonism^14,71^. Consistently we recently reported that both the intrinsic excitability and thalamocortical transmission were selectively downregulated in PTNs, but not ITNs, using mice with 6-hydroxydopamine lesions^8,11^. Further analysis indicated that postsynaptic NMDA receptors of pyramidal tract neurons are critical in mediating cortical circuit adaptations in parkinsonism. In the present study, we further demonstrated that a partial loss of striatal dopamine did not affect the intrinsic excitability and thalamocortical transmission of the corticospinal neurons (Fig. 8). These data suggest that striatal dopamine loss has to be greater than a certain threshold to trigger NMDA receptor-mediated cortical circuit adaptations. A gradual development of the synchronized bursting pattern of activity throughout the basal ganglia-thalamocortical network might be a key network determinant in mediating an effective stimulation of postsynaptic NMDA receptors in cortical pyramidal neurons^72^. Of particular interest, the emergence of the synchronized bursting pattern of network activity involves neural plasticity processes associated with chronic and robust dopamine depletion in the striatum^42,73,74^. Thus, the motor cortex may exhibit secondary circuit adaptations at late stages of nigrostriatal dopamine neurodegeneration due to pathological basal ganglia outputs to the motor thalamus.

αSyn pathology may reach the cerebral cortex at the Braak stage 4 and beyond^17,18^. It is important to study whether αSyn pathology and nigral dopaminergic degeneration interact to disrupt the integrity and function of the cortical circuits in PD. Our work presented preliminary data as an initial attempt to address this question. Detailed analysis of cortical circuits in the presence of both severe dopamine depletion and Lewy-like pathology remains needed to advance our understanding of cortical circuit operation under parkinsonian state.

## Supplementary information

Supplementary figures

## Data Availability

The datasets used and/or analyzed during the current study have been deposited and are publicly available from 10.5281/zenodo.14057740.
