# Supplementary material for: Motor cortical neuronal hyperexcitability associated with α-synuclein aggregation
Source: NPJ Parkinsons Dis. 2025 Jan 15;11:18. doi: 10.1038/s41531-024-00867-z (PMC11733020; doi:10.1038/s41531-024-00867-z)
Supplement: Supplementary file 1 — Supplementary figures [file 41531_2024_867_MOESM1_ESM.docx]

**
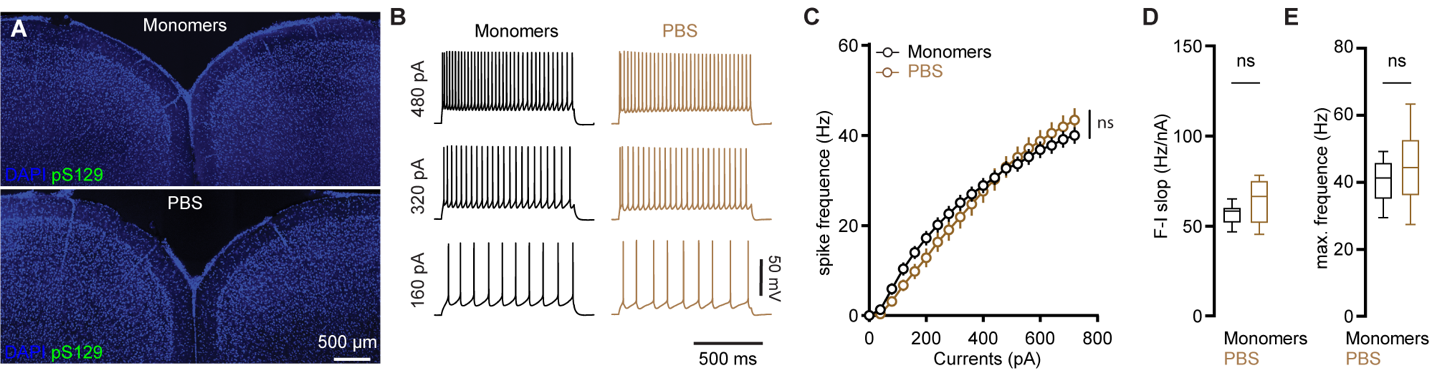
**

**Supplemental Figure 1. No difference in neuronal excitability of M2 CSNs between PBS- and αSyn monomer-injected mice. A)** Confocal images showing the absence of pS129 immunoreactive αSyn pathology in the motor cortex of PBS and monomer-injected mice. **B**) AP spike trains of M2 CSNs from PBS- and monomer-injected mice in response to different levels of somatic current injections (i.e., 160 pA, 320 pA, and 480 pA for 1 sec). **C-E**) frequency-current curves (C) and box plots (D-E) showing no changes in the intrinsic excitability of CSNs between PBS- and monomer-injected mice. F-I slopes monomers = 58.25 [51.68, 60.38] Hz/nA, n = 12 neurons/2 mice; PBS = 66.40 [51.55, 75.18] Hz/nA, n = 22 neurons/3 mice. *p* = 0.1, MWU. Maximal firing frequency, monomers = 41.00 [34.75, 45.50] Hz/nA, n = 12 neurons/2 mice; PBS = 44.00 [35.75, 52.25], n = 22 neurons/3 mice, *p* = 0.4169, MWU.

**
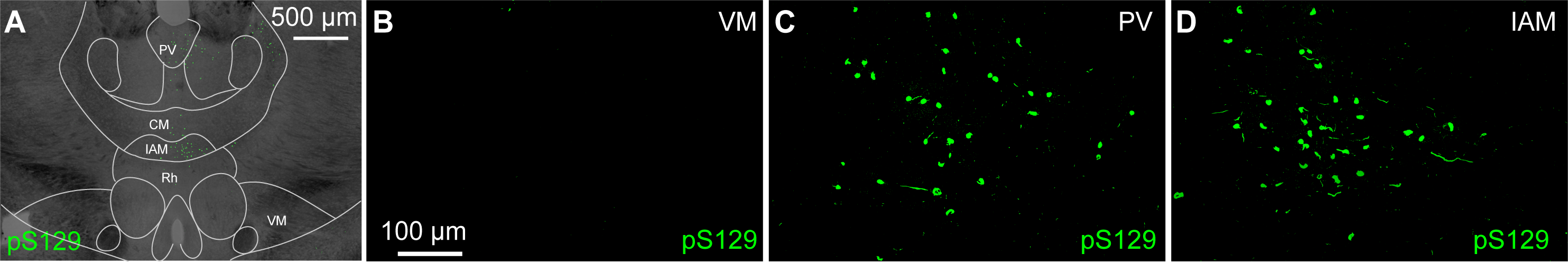
**

**Supplemental Figure 2.** Representative confocal image showing the absence of pS129 αSyn pathology in subregions of the thalamus. No cytoplasmic pS129-ir αSyn aggregates were detected in the ventromedial (VM, B) thalamus. Mild to moderate levels of αSyn pathology were detected in the periventricular (PV, C), interanteromedial (IAM, D) subregions of the thalamus.

**
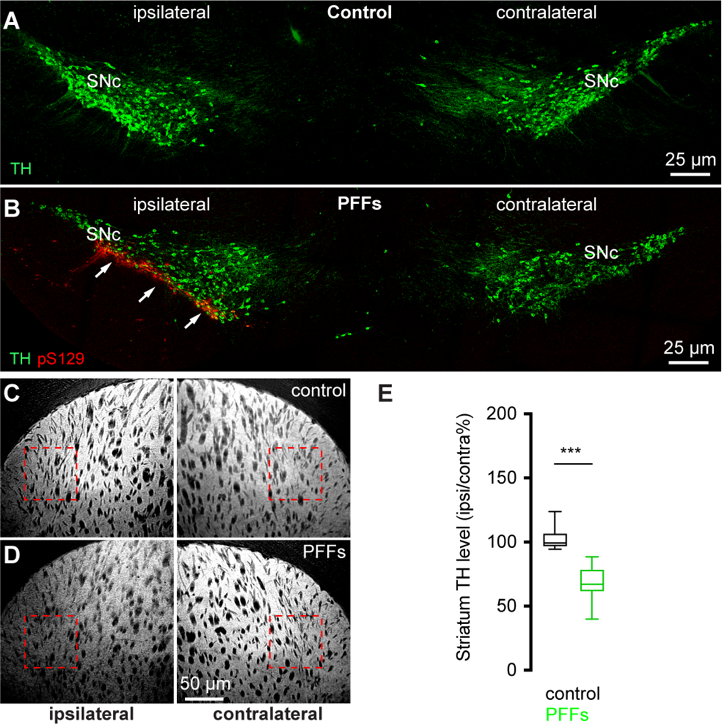
**

**Supplemental Figure 3. Intrastriatal PFFs injection induces nigrostriatal degeneration. (A and B)** Representative confocal images showing the expression of TH-expressing cells and pS129 αSyn pathology in the bilateral SNc of controls and PFFs-injected mice at 3 mpi. Arrows indicate the formation of pS129 αSyn aggregates in the ventral tier of the SNc ipsilateral to the PFFs-injected striatum. **C-D**) Representative confocal images showing the TH expression in the dorsal striatum of control and PFFs-injected mice at 3 mpi. Red boxes indicated the region of interest for TH quantification. **E**) Box plots showing a significant reduction of TH immunoreactivity in the striatum receiving PFFs injections. Data represent TH immunoreactivity from the ipsilateral striatum normalized to the contralateral striatum of both controls and PFFs-injected mice (ipsi/contra striatal TH%, controls = 99.42, [96.54, 106.9]%, PFFs = 67.01 [61.52, 78.81]%, n = 22 slices/11 mice for each group, *p* < 0.0001, MWU).
